# Supplementary figures and images for: IGHMBP2 deletion suppresses translation and activates the integrated stress response
Source: Life Sci Alliance. 2024 May 21;7(8):e202302554. doi: 10.26508/lsa.202302554 (PMC11109757; doi:10.26508/lsa.202302554)

Fig. 1C

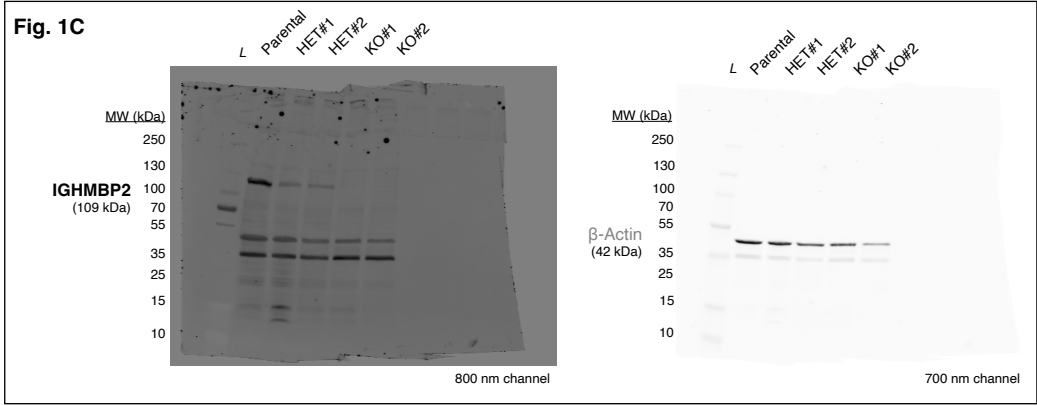

L: PageRuler Plus  
protein ladder

Supplement: Supplementary file 1 [file LSA-2023-02554_SdataF1.pdf]

Fig. 2B

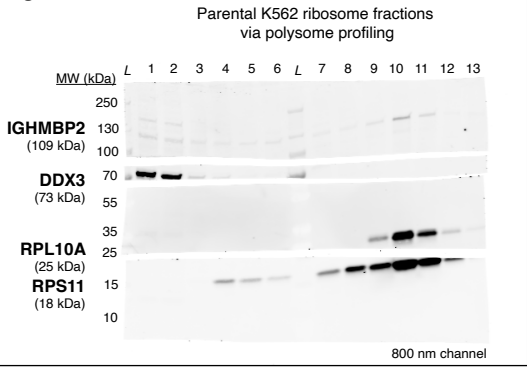

L: PageRuler Plus  
protein ladder

Supplement: Supplementary file 2 [file LSA-2023-02554_SdataF2.pdf]

**Fig. S9A**

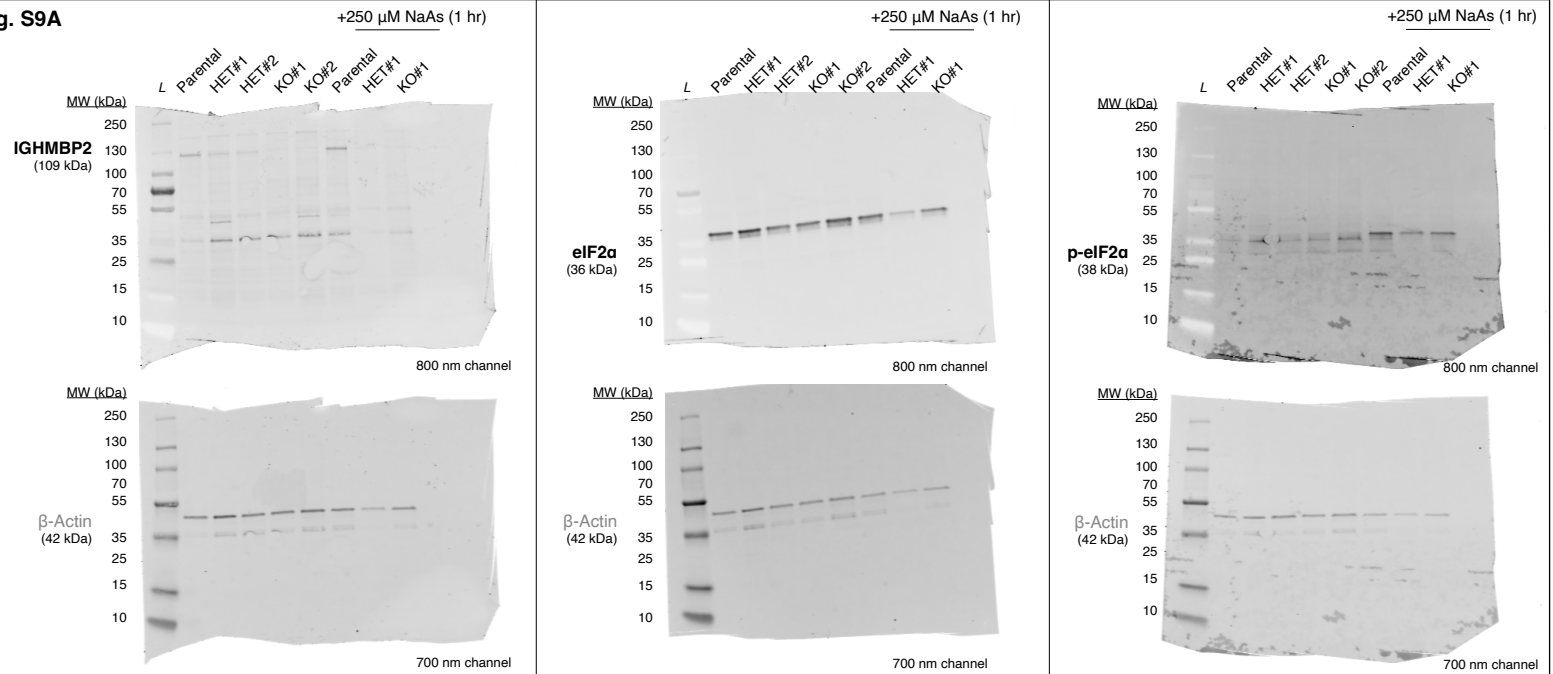

**Fig. S9B**

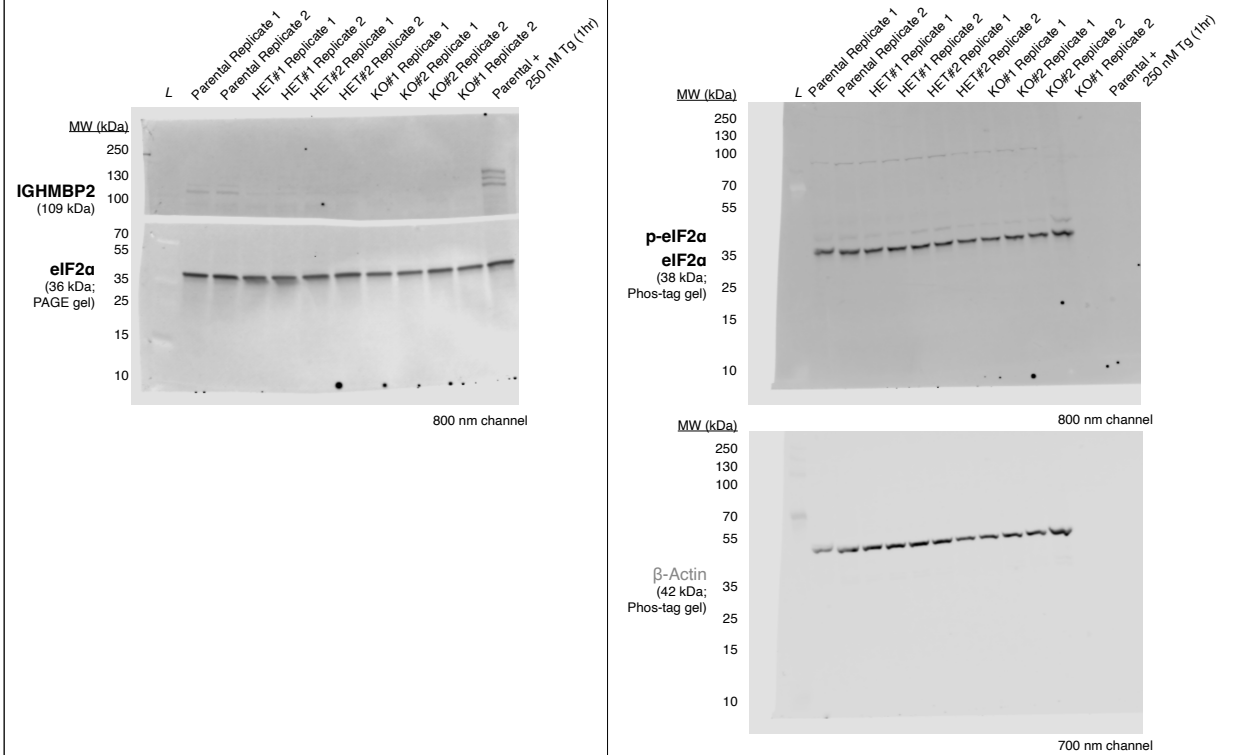

L: PageRuler Plus protein ladder  
NaAs: Sodium arsenite  
Tg: Thapsigargin

Supplement: Supplementary file 5 [file LSA-2023-02554_SdataFS9.pdf]

Fig. S12B

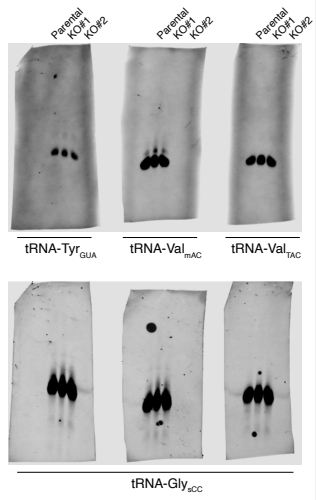

Supplement: Supplementary file 6 [file LSA-2023-02554_SdataFS12.pdf]
